# Performance of name-to-gender inference: comparison between Gender API, NamSor, and genderize.io in a multicultural global dataset

**DOI:** 10.1007/s11739-026-04334-w

**Published:** 2026-04-03

**Authors:** Paul Sebo, Amrollah Shamsi, Ting Wang

**Affiliations:** 1 University Institute for Primary Care (IuMFE), University of Geneva, 1211 Geneva, Switzerland; 2 Clinical Research Development Center, The Persian Gulf Martyrs Hospital, Bushehr University of Medical Sciences, Bushehr, Iran; 3 School of Library and Information Management, Emporia State University, Emporia, KS USA

**Keywords:** Bibliometrics, Evaluation, Gender, Gender API, Gender detection tool, Genderize, Geographic bias, Misclassification, Multicultural, Name-to-gender inference, NamSor, Performance

## Abstract

Inferring gender from first names is widely used in medical research when gender data are unavailable, but tool performance may vary across cultural and linguistic contexts. We compared the real-world performance of three name-to-gender inference tools (Gender API, NamSor, and genderize.io) in a large multicultural dataset with self-reported gender labels. We assembled publicly available results from seven major 2025 marathons: New York, Berlin, Paris, Shanghai, Tokyo, Dubai, and Abu Dhabi (*n* = 11,999, women = 6,000, men = 5,999). First names, with and without country of nationality, were submitted to each tool. We computed confusion matrices and performance metrics: overall error (errorCoded: misclassifications + nonclassifications), misclassifications among classified names (errorCodedWithoutNA), and nonclassifications (naCoded). A priori, a tool was defined as accurate when errorCoded was < 10%. Pairwise tool comparisons used McNemar’s test. We also assessed confidence-threshold subsetting (≥ 60%– ≥ 90%) and region-specific performance. All three tools met the prespecified accuracy criterion overall. NamSor outperformed both comparators (*p* < 0.001). Without country information, errorCoded was 0.0481 (NamSor), 0.0867 (Gender API), and 0.0798 (genderize.io); NamSor produced no unclassified names. Performance was substantially lower for Asian nationalities and improved after excluding Asia/China. Increasing confidence thresholds reduced misclassifications but increased nonclassifications, raising overall error. Adding country improved performance for Gender API (*p* < 0.001) and genderize.io (*p* = 0.03), but not for NamSor (*p* = 0.41). In Conclusion, although all tools were accurate overall, performance depended strongly on geographic composition. Researchers should report expected misclassification rates based on external validation studies, justify confidence thresholds, and rely on tools validated in culturally comparable populations.

**Supplementary Information:**

The online version contains supplementary material available at https://doi.org/10.1007/s11739-026-04334-w.

## Introduction

Inferring gender from first names has become a widely adopted approach in biomedical research, bibliometrics, and social science investigations, particularly when large databases lack explicit gender information [1]. Automated name-to-gender inference services, also referred to as gender detection tools, provide a rapid and cost-effective method to enrich datasets, which explains their increasing use in medical studies examining gender disparities in academic authorship [2–12], editorial leadership [13–19], grant allocation [20–22], and scientific communication [23–28]. These tools are now routinely integrated into analyses of equity in medicine, making their accuracy a central methodological concern.

Despite their convenience, the performance of these tools varies markedly depending on the linguistic and cultural diversity of the population studied [1, 29–33]. In our previous Swiss-based study, we found that Gender API, NamSor, genderize.io, and Wiki-Gendersort showed low misclassification rates but substantial differences in nonclassification rates, particularly for genderize.io, which demonstrated a high proportion of missing assignments [1]. Interestingly, a subsequent study reported that genderize.io did not produce particularly high proportions of unclassified names [30], which may indicate that performance varies across countries depending on the distribution of first names present in a given population. Although Switzerland is a multilingual and multicultural environment, its particular mix of linguistic and cultural backgrounds may differ substantially from other Western countries. More broadly, previous research has shown that name-to-gender inference tools tend to be less accurate for names of non-Western origin, especially those from Asia [29, 30, 32, 33]. These observations indicate that generalizing accuracy metrics derived from a single population may lead to overly optimistic expectations of tool performance.

In addition, with the exception of our previous Swiss-based validation study [1], published evaluations of gender detection tools have relied on datasets in which “true” gender was not self-reported but indirectly ascertained using name-based, institutional, or web-derived proxies [29–33]. This methodological circularity limits the validity of reported accuracy estimates, because tool performance is not evaluated against verified gender labels but against proxy classifications that may share similar cultural and linguistic biases.

To address these limitations, we designed a validation study in a culturally and linguistically heterogeneous population providing verified self-reported gender labels. International marathon datasets are particularly suitable for this purpose, as they encompass participants from diverse national backgrounds and often publish first name, surname, self-reported gender, and country of origin. The availability of verified gender information makes these datasets uniquely appropriate for evaluating inference tools in real-world conditions. In this study, we extracted results from seven major marathons, resulting in a large and internationally representative sample.

Using this global dataset, the objective of this study was to compare the performance of three widely used gender detection tools, i.e., Gender API, NamSor, and genderize.io. The analysis assessed their ability to correctly classify gender, quantified errors and nonclassifications, and evaluated differences in performance. This investigation represents, to our knowledge, the first large-scale evaluations of name-to-gender inference tools using an internationally diverse dataset with verified gender labels.

Because name-to-gender inference tools are increasingly used in general internal medicine research and other clinical epidemiology studies when gender data are unavailable, reliable real-world accuracy estimates are essential. This study provides evidence-based guidance to inform methodological choices when gender inference is applied to multicultural datasets.

## Methods

### Study design and data sources

This comparative accuracy study used publicly available finisher data from six major international marathons held in 2025: New York, Berlin, Paris, Shanghai, Tokyo, and Dubai. Each event publishes first name, surname, nationality, self-reported gender, and finishing position. For each marathon, the first 1,000 female and the first 1,000 male finishers were included. For the Dubai marathon, only 381 female finishers were available; therefore, the female cohort was completed with the first 619 female finishers from the Abu Dhabi marathon. This resulted in a dataset of twelve thousand individuals from a wide range of cultural and linguistic backgrounds. The final analytical dataset included 11,999 participants, as one male finisher from the Dubai marathon had missing first/last name information and was therefore excluded.

All names were extracted exactly as presented on the official marathon websites, without modifications to spelling, hyphenation, or diacritics, except that they were converted to lowercase for standardization. In the case of the Shanghai marathon, one of the authors (TW) translated Chinese names originally written in Chinese characters into their Romanized form using standard pinyin conventions, so that all tools could process names consistently across datasets.

For New York, Shanghai, and Tokyo, first names and surnames were provided together on a single line; in these cases, the first token was extracted as the first name and the remaining string was considered the surname.

### Gender detection tools and submitted variables

Three gender detection tools frequently used in research were evaluated: Gender API [34], NamSor [35], and genderize.io [36]. Although Wiki-Gendersort was included in our original Swiss-based study [1], it was not examined in the present analysis because an internet search showed that it is not used in research.

Gender API accepts CSV or Excel files with first name and, when applicable, country of nationality/origin (free tier: 100 inferences/month). NamSor accepts CSV, Excel, or text files with first name, optionally surname and country of nationality/origin (free tier: 2,500 inferences/month). Genderize.io accepts CSV files with first name and, when applicable, country of nationality/origin (free tier: 100 inferences/day).

Analyses were performed using first name with and without country of nationality for Gender API and genderize.io, and using first name and surname with and without country of nationality for NamSor. This approach enabled each tool to operate under its own optimal configuration while preserving comparability across services. It was prespecified that when no inference was returned, the query was repeated by swapping the first name and surname fields (i.e., submitting the surname in place of the first name).

For each tool, we retrieved the predicted gender and the confidence score associated with the prediction. The terminology used for the confidence score differs across services: Gender API provides probability values expressed as percentages up to 100% (“accuracy”), whereas NamSor and genderize.io return probability values up to 1 ("calibrated probability" and “probability”, respectively). All these values represent a measure of confidence and are referred to in this manuscript as “confidence level” or “confidence score”.

Data extraction from marathon websites was performed in December 2025, and submissions to gender detection tools were conducted in January 2026.

### Outcome measures and performance metrics

The predicted gender was compared with the self-reported gender provided by marathon organizers. Confusion matrices were constructed to capture correct classifications, misclassifications, and nonclassifications.

Following previously published methodological frameworks, several established performance metrics were computed [1, 29, 31, 33, 37]. The overall proportion of errors (errorCoded) included both misclassifications and nonclassifications. The proportion of incorrect predictions among classified names (errorCodedWithoutNA) reflected pure misclassifications. The proportion of names that the tool failed to classify (naCoded) measured nonclassifications. A directional indicator (errorGenderBias) was also computed to evaluate whether a tool tended to overassign one gender over the other. These complementary indicators allowed for a detailed assessment of performance, and the formulas used to calculate each metric are summarized in Box 1.

**Box 1:** Performance metrics used in the study

Metric	Definition	Formula^1^
errorCoded	Overall error rate, including misclassifications and nonclassifications	errorCoded = (fm + mf + fu + mu)/(ff + fm + mf + mm + fu + mu)
errorCodedWithoutNA	Misclassification rate among names for which a classification was provided	errorCodedWithoutNA = (fm + mf)/(ff + fm + mf + mm)
naCoded	Proportion of names not classified by the tool	naCoded = (fu + mu)/(ff + fm + mf + mm + fu + mu)
errorGenderBias	Directional bias toward overassigning one gender	errorGenderBias = (mf − fm)/(ff + fm + mf + mm)

^1^ In these formulas, ff and mm correspond to correct classifications, fm and mf correspond to misclassifications, and fu and mu correspond to nonclassifications (“unknown”)

Because there is no universally accepted cutoff to define when a gender detection tool is sufficiently accurate, we prespecified an operational criterion, consistent with our previous work using error-based metrics: a tool was considered accurate when errorCoded was < 10% [33]. This threshold was chosen a priori as a pragmatic benchmark to support interpretation and does not represent an external standard.

### Confidence-based data subsetting

To examine tool performance under varying confidence thresholds, we restricted the dataset to predictions meeting or exceeding specific confidence levels. Confidence scores were used to create subsamples corresponding to accuracy thresholds of 60%, 70%, 80%, and 90%. Each subset contained only predictions at or above the chosen confidence value. This approach made it possible to assess how performance changes when researchers opt to rely only on higher-confidence predictions, a strategy increasingly used in bibliometric studies that employ gender inference tools.

### Statistical analyses

Pairwise comparisons between gender detection tools and between models with and without country information were performed using McNemar’s test on paired individual-level classifications. All analyses were conducted using Stata version 15 (StataCorp, College Station, Texas, USA).

### Ethical considerations

Since this study did not involve the collection of personal health-related data, it did not require ethical review according to current Swiss law. All information was obtained from publicly accessible marathon result pages, and the dataset was fully anonymized before being added as supplementary material.

## Results

### Study sample and nationality distribution

The dataset included 11,999 marathon finishers, with 6,000 women and 5,999 men. Participants represented a wide range of nationalities (SM Table 1, *n* = 137 countries). The most frequent nationalities were China (*n* = 2,052; 17.1%), the United States (*n* = 1,863; 15.5%), France (*n* = 1,616; 13.5%), Japan (*n* = 1,149; 9.6%), and the United Kingdom (*n* = 955; 8.0%).

### Confusion matrices

Confusion matrices for each tool are shown in Table 1. NamSor produced no unclassified names, both with and without country information. Gender API and genderize.io generated small proportions of unclassified names without country information, which increased slightly for genderize.io when country information was provided.

**Table 1:** Confusion matrices of NamSor, Gender API, and genderize.io for gender classification in the marathon cohort (*n* = 11,999), with and without country information for gender assignment

Condition	Classified as women, *n* (%)	Classified as men, *n* (%)	Unclassified, *n* (%)
Without using country information			
NamSor			
Female individuals (*n* = 6,000)	5,695 (94.9)	305 (5.1)	0
Male individuals (*n* = 5,999)	272 (4.5)	5,727 (95.5)	0
Gender API			
Female individuals (*n* = 6,000)	5,262 (87.7)	723 (12.1)	15 (0.3)
Male individuals (*n* = 5,999)	280 (4.7)	5,697 (94.9)	22 (0.4)
Genderize.io			
Female individuals (*n* = 6,000)	5,434 (90.6)	562 (9.4)	4 (0.1)
Male individuals (*n* = 5,999)	380 (6.3)	5,608 (93.5)	11 (0.2)
Using country information			
NamSor			
Female individuals (*n* = 6,000)	5,699 (95.0)	301 (5.0)	0
Male individuals (*n* = 5,999)	272 (4.5)	5,727 (95.5)	0
Gender API			
Female individuals (*n* = 6,000)	5,358 (89.3)	626 (10.4)	16 (0.3)
Male individuals (*n* = 5,999)	234 (3.9)	5,737 (95.6)	28 (0.5)
Genderize.io			
Female individuals (*n* = 6,000)	5,443 (90.7)	520 (8.7)	37 (0.6)
Male individuals (*n* = 5,999)	309 (5.2)	5,653 (94.2)	37 (0.6)

Comparison between NamSor, Gender API, and genderize.io (McNemar’s test): *p*-value < 0.001

### Overall performance metrics in the full sample

Overall performance metrics are shown in Table 2. Without country information, errorCoded was lowest for NamSor (0.0481), followed by genderize.io (0.0798) and Gender API (0.0867). Nonclassification was negligible for all three tools (≤ 0.3%).

**Table 2:** Performance metrics of NamSor, Gender API, and genderize.io in the marathon cohort (*n* = 11,999), with and without country information for gender assignment

Condition	errorCoded	errorcodedWithoutNA	naCoded	errorGenderBias
Without using country information				
Namsor	0.0481	0.0481	0	−0.0028
Gender API	0.0867	0.0838	0.0031	−0.0370
Genderize.io	0.0798	0.0786	0.0013	−0.0152
Using country information				
NamSor	0.0478	0.0478	0	−0.0024
Gender API	0.0753	0.0719	0.0037	−0.0328
Genderize.io	0.0753	0.0695	0.0062	−0.0177

With country information, errorCoded decreased for all three tools (NamSor: 0.0478; Gender API: 0.0753; genderize.io: 0.0753), while nonclassification increased for Gender API and genderize.io. NamSor showed minimal directional bias, whereas Gender API and genderize.io tended to overassign men. Using the prespecified accuracy criterion (errorCoded < 10%), all three tools were accurate in the overall sample, both with and without country information.

### Performance across confidence thresholds

Across tools, increasing the confidence threshold was associated with a progressive increase in the overall error rate (errorCoded), driven by a marked rise in the proportion of unclassified predictions (naCoded), while the misclassification rate among classified names (errorCodedWithoutNA) decreased (SM Tables 2–4). For example, without country information, NamSor errorCoded increased from 0.0481 in the full sample to 0.2171 at the ≥ 90% threshold, while errorCodedWithoutNA decreased from 0.0481 to 0.0108, reflecting fewer misclassifications among retained predictions but many more unclassified names. Similar patterns were observed for Gender API and genderize.io.

### Sensitivity analyses excluding Asia and China

Tool performance improved substantially after excluding participants from Asia and, more specifically, after excluding participants from China (SM Tables 2–4). For NamSor, errorCoded decreased to 0.0067 when excluding Asia and to 0.0100 when excluding China in the configuration without country information; the corresponding values with country information were 0.0065 and 0.0095. A similar pattern was observed for Gender API and genderize.io.

### Performance by region of nationality

Marked heterogeneity was observed across regions (SM Table 5). For all three tools, performance was substantially lower in Asia than in Europe or the Americas. Without country information, errorCoded was 0.1279 vs 0.0036 for NamSor, 0.1860 vs 0.0238 for Gender API, and 0.1728 vs 0.0240 for genderize.io (Asia vs Europe). Similar patterns were observed when country information was used. ErrorGenderBias was most pronounced for Gender API in Asia (− 0.0857 without country; − 0.0827 with country), consistent with overassignment of men in that region.

### Performance by country of nationality

Country-level analyses, restricted to countries with at least 20 participants, revealed marked between-country variability (SM Tables 6–8). Across tools, the poorest performance was observed primarily in countries from Asia. For NamSor, the highest error rates were observed in Singapore (errorCoded = 0.3000), China (0.2329), South Korea (0.1081), Malaysia (0.0952), and the Philippines (0.0866). For Gender API, the poorest performance was observed in Hong Kong (0.4038), Singapore (0.4000), Taiwan (0.2973), Ethiopia (0.2813), and China (0.2778). For genderize.io, the highest error rates were observed in Singapore (0.5000), Hong Kong (0.3654), China (0.2597), Malaysia (0.2381), and Taiwan (0.1892).

### Statistical comparison between and within tools

Pairwise McNemar tests showed that, without country information, NamSor achieved higher classification accuracy than genderize.io (95.19% vs 92.02%) and Gender API (95.19% vs 91.33%) (both *p* < 0.001), and that genderize.io slightly outperformed Gender API (*p* = 0.001). With country information, NamSor again outperformed both genderize.io and Gender API (both 95.22% vs 92.47%; *p* < 0.001), while no significant difference was observed between genderize.io and Gender API (*p* = 0.97).

Within-tool comparisons showed that adding country information did not change NamSor performance (*p* = 0.41), but slightly improved classification accuracy for genderize.io (*p* = 0.026) and more markedly for Gender API (*p* < 0.001).

## Discussion

### Summary of key findings

Several key findings emerge in this study evaluating the real-world performance of three widely used name-to-gender inference tools (NamSor, Gender API, and genderize.io) in a large, multicultural dataset with self-reported gender labels. First, all three tools fulfilled the prespecified accuracy criterion in the overall sample, but NamSor consistently achieved the highest classification accuracy. Second, tool performance depended strongly on geographic composition, with markedly lower accuracy in several Asian countries. Overall performance improved substantially after excluding Asian participants, and particularly after excluding China, which was the most represented nationality in the cohort. Third, increasing confidence thresholds reduced misclassifications but increased nonclassifications, resulting in higher overall error. Finally, providing country information improved performance for Gender API and genderize.io but had minimal impact on NamSor.

### Comparison with existing literature

Our findings both corroborate and refine recent benchmarking studies of name-to-gender inference tools. In our prior Swiss-based validation study (*n* = 6,131; 50.3% women; self-reported gender), conducted among physicians practicing in Switzerland, NamSor and Gender API demonstrated substantially lower overall error rates than those observed in the present multinational cohort (NamSor errorCoded = 0.0202; Gender API errorCoded = 0.0181) [1]. In contrast, genderize.io showed markedly higher overall error in that study (errorCoded = 0.1766), driven by a high nonclassification rate. That cohort was culturally and linguistically more homogeneous, with the majority of physicians originating from Western Europe, and names were submitted in their original formats without normalization. The generally superior performance observed in that setting supports our present finding that accuracy estimates derived from Western populations substantially underestimate error when applied to multicultural datasets. Interestingly, nonclassification rates for genderize.io were considerably lower in the present study, suggesting improvements since 2021, potentially related to expanded name coverage or improved batch-processing capabilities.

Domínguez et al. (2024; *n* = 5,779; 34.1% women; gender not self-determined) evaluated NamSor and Gender API using a bibliometric dataset originally compiled by Santamaría and Mihaljević (2018) from multiple scientific sources (zbMATH, genderizeR, PubMed, and Web of Science) [29, 31]. In that dataset, author gender had been manually assigned through extensive web-based searches of institutional pages, biographies, photographs, and other public records. The cohort comprised authors from 113 countries, with 61% of names of European origin, 34% Asian, and 5% African. NamSor and Gender API both met the <10% error threshold in that study (errorCoded = 0.070 and 0.072, respectively).

Van Helden et al. (2024; *n* = 32,968; 34.6% women; gender not self-determined) evaluated genderize.io and Gender API in a gold-standard registry of cancer clinical trialists sourced from the HemOnc knowledge base, which aggregates author records primarily from MEDLINE-indexed oncology and hematology trials [30]. Names underwent extensive normalization to address diacritics, misspellings, multipart surnames, and related variations. Binary gender was manually assigned using a combination of automated forename mapping, web-based searches, and consensus determinations, with country affiliations also normalized. In that study, genderize.io and Gender API achieved high overall classification accuracy (96.6% and 96.1% correct, respectively), with low nonclassification rates (1.1% for both tools).

Taken together, these comparisons demonstrate that reported tool performance is strongly dependent on population composition, cultural diversity, normalization practices, and the nature of the gender reference standard. In particular, accuracy estimates derived from homogeneous Western cohorts may substantially overestimate real-world performance in culturally diverse populations.

The reduced accuracy for Chinese and other East Asian names likely reflects several linguistic and algorithmic factors. Many given names in these languages are gender-neutral and lack morphological markers of gender, which limits the ability of inference models trained primarily on Western naming conventions to distinguish male from female names. In addition, distinct Chinese given names may share identical Romanized spellings despite being written with different characters, some of which are preferentially used for males and others for females. The homophony, combined with variable transliteration practices, further reduces the discriminatory power of algorithms that rely on Romanized input alone.

### Implications for practice and research

Name-to-gender inference tools are now routinely used in medical research focused on gender inequalities, in which reporting of the studied population, tool configuration, applied confidence thresholds, and handling of unclassified names is essential. Our findings demonstrate that these methodological choices have major consequences for estimated gender distributions and observed disparities, particularly in culturally diverse datasets.

Restricting analyses to predictions exceeding predefined confidence thresholds is commonly intended to improve classification reliability. Our results show that while this practice reduces misclassifications among classified names, it substantially increases nonclassifications and leads to higher overall error rates. This approach therefore introduces systematic selection bias by disproportionately excluding individuals from regions in which tool confidence is lower, especially Asian countries. Consequently, threshold-based filtering may underrepresent non-Western populations and distort estimates of gender disparities.

General internal medicine researchers and clinical epidemiologists increasingly rely on name-to-gender inference when working with large datasets that lack self-reported gender. Our findings provide evidence-based guidance to inform methodological choices in multicultural datasets, including tool selection, whether to incorporate country information, the consequences of applying confidence thresholds, and transparent reporting of nonclassifications. We also encourage region-specific sensitivity analyses when international data are analyzed.

### Strengths and limitations

This study has several strengths, including the use of a large, balanced dataset with verified self-reported gender, inclusion of participants from a wide range of national backgrounds, and systematic evaluation of three widely used tools across multiple analytic configurations.

However, several limitations must be acknowledged. First, marathon participants are not necessarily representative of the general population; names may differ in distribution by age, socioeconomic status, and cultural subgroups. Second, gender inference was restricted to binary classifications and does not capture nonbinary or gender-diverse identities, which limits applicability in contexts where more inclusive gender data are required. Third, romanization of non-Latin scripts, particularly Chinese names, may introduce additional uncertainty, although this reflects real-world research conditions faced by most bibliometric studies. Fourth, some countries were represented by relatively small sample sizes, limiting the precision of country-specific estimates. Fifth, performance metrics may vary over time as vendors update their underlying databases, meaning that reported accuracies may not remain stable. Sixth, confidence scores provided by the tools are proprietary and may not be directly comparable across services. Finally, because “accuracy” lacks a universal definition in this field, our prespecified operational cutoff may not generalize to all research contexts, and alternative thresholds could yield different conclusions.

## Conclusion

Using a prespecified benchmark (errorCoded < 10%), NamSor, Gender API, and genderize.io all met the accuracy criterion in the overall marathon cohort. However, tool performance varied markedly by region of nationality, with substantially higher error rates in several Asian countries. These findings indicate that accuracy estimates derived from homogeneous Western populations may substantially overestimate real-world performance in multicultural datasets.

By providing large-scale validation using self-reported gender as the ground-truth reference across multiple continents, this study establishes a practical reference framework for selecting and configuring gender inference tools in international medical research.

## Supplementary Information

Below is the link to the electronic supplementary material.

Supplementary file1 (DOCX 36 KB)

Supplementary file2 (XLS 3438 KB)

## Data Availability

The data associated with this article are available in the Supplementary Material.
