# Supplementary material for: Performance of name-to-gender inference: comparison between Gender API, NamSor, and genderize.io in a multicultural global dataset
Source: Intern Emerg Med. 2026 Apr 3;21(5):1585–92. doi: 10.1007/s11739-026-04334-w (PMC13672574; doi:10.1007/s11739-026-04334-w)
Supplement: Supplementary file 1 [file 11739_2026_4334_MOESM1_ESM.docx]

Supplementary Material

SM Table 1. Distribution of participants by country of nationality in the marathon cohort, ranked by number of participants (n = 11,999 participants and 137 countries).

| Country | n | % | Cumulative % |
| --- | --- | --- | --- |
| China | 2,052 | 17.10 | 17.10 |
| United States | 1,863 | 15.53 | 32.63 |
| France | 1,616 | 13.47 | 46.10 |
| Japan | 1,149 | 9.58 | 55.67 |
| United Kingdom | 955 | 7.96 | 63.63 |
| Germany | 446 | 3.72 | 67.35 |
| Philippines | 231 | 1.93 | 69.27 |
| Canada | 217 | 1.81 | 71.08 |
| Russia | 189 | 1.58 | 72.66 |
| Spain | 166 | 1.38 | 74.04 |
| Australia | 155 | 1.29 | 75.33 |
| Brazil | 155 | 1.29 | 76.62 |
| Italy | 155 | 1.29 | 77.91 |
| Mexico | 150 | 1.25 | 79.16 |
| Ireland | 148 | 1.23 | 80.40 |
| South Africa | 133 | 1.11 | 81.51 |
| Netherlands | 128 | 1.07 | 82.57 |
| Norway | 122 | 1.02 | 83.59 |
| Poland | 117 | 0.98 | 84.57 |
| India | 100 | 0.83 | 85.40 |
| Ethiopia | 96 | 0.80 | 86.20 |
| Belgium | 94 | 0.78 | 86.98 |
| Switzerland | 81 | 0.68 | 87.66 |
| Kenya | 76 | 0.63 | 88.29 |
| Iran | 71 | 0.59 | 88.88 |
| United Arab Emirates | 71 | 0.59 | 89.47 |
| Sweden | 64 | 0.53 | 90.01 |
| Colombia | 61 | 0.51 | 90.52 |
| Denmark | 56 | 0.47 | 90.98 |
| Hong Kong | 52 | 0.43 | 91.42 |
| Austria | 46 | 0.38 | 91.80 |
| Kazakhstan | 41 | 0.34 | 92.14 |
| Portugal | 40 | 0.33 | 92.47 |
| South Korea | 37 | 0.31 | 92.78 |
| Taiwan | 37 | 0.31 | 93.09 |
| Argentina | 33 | 0.28 | 93.37 |
| New Zealand | 31 | 0.26 | 93.62 |
| Chile | 29 | 0.24 | 93.87 |
| Lithuania | 29 | 0.24 | 94.11 |
| Finland | 28 | 0.23 | 94.34 |
| Greece | 27 | 0.23 | 94.57 |
| Israel | 25 | 0.21 | 94.77 |
| Romania | 25 | 0.21 | 94.98 |
| Czech Republic | 24 | 0.20 | 95.18 |
| Egypt | 24 | 0.20 | 95.38 |
| Lebanon | 24 | 0.20 | 95.58 |
| Morocco | 24 | 0.20 | 95.78 |
| Costa Rica | 23 | 0.19 | 95.97 |
| Slovak Republic | 22 | 0.18 | 96.16 |
| Malaysia | 21 | 0.18 | 96.33 |
| Hungary | 20 | 0.17 | 96.50 |
| Singapore | 20 | 0.17 | 96.67 |
| Ukraine | 20 | 0.17 | 96.83 |
| Indonesia | 19 | 0.16 | 96.99 |
| Estonia | 17 | 0.14 | 97.13 |
| Turkey | 17 | 0.14 | 97.27 |
| Thailand | 15 | 0.13 | 97.40 |
| Jordan | 14 | 0.12 | 97.52 |
| Peru | 13 | 0.11 | 97.62 |
| Guatemala | 11 | 0.09 | 97.72 |
| Nepal | 11 | 0.09 | 97.81 |
| Uganda | 11 | 0.09 | 97.90 |
| Vietnam | 11 | 0.09 | 97.99 |
| Venezuela | 10 | 0.08 | 98.07 |
| Iceland | 9 | 0.08 | 98.15 |
| Syria | 9 | 0.08 | 98.22 |
| Zimbabwe | 9 | 0.08 | 98.30 |
| Ecuador | 8 | 0.07 | 98.37 |
| Latvia | 8 | 0.07 | 98.43 |
| Yugoslavia | 8 | 0.07 | 98.50 |
| Croatia | 7 | 0.06 | 98.56 |
| Pakistan | 7 | 0.06 | 98.62 |
| Tanzania | 7 | 0.06 | 98.67 |
| Algeria | 6 | 0.05 | 98.72 |
| Azerbaijan | 6 | 0.05 | 98.77 |
| Malta | 6 | 0.05 | 98.82 |
| Slovenia | 6 | 0.05 | 98.87 |
| Armenia | 5 | 0.04 | 98.92 |
| Bulgaria | 5 | 0.04 | 98.96 |
| Dominican Republic | 5 | 0.04 | 99.00 |
| Kyrgyz Republic | 5 | 0.04 | 99.04 |
| Luxembourg | 5 | 0.04 | 99.08 |
| Belarus | 4 | 0.03 | 99.12 |
| Botswana | 4 | 0.03 | 99.15 |
| Oman | 4 | 0.03 | 99.18 |
| Palestine | 4 | 0.03 | 99.22 |
| Panama | 4 | 0.03 | 99.25 |
| Saudi Arabia | 4 | 0.03 | 99.28 |
| Sudan | 4 | 0.03 | 99.32 |
| Tunisia | 4 | 0.03 | 99.35 |
| Uruguay | 4 | 0.03 | 99.38 |
| Cyprus | 3 | 0.03 | 99.41 |
| Kuwait | 3 | 0.03 | 99.43 |
| Macao | 3 | 0.03 | 99.46 |
| Mauritius | 3 | 0.03 | 99.48 |
| Moldova | 3 | 0.03 | 99.51 |
| Mongolia | 3 | 0.03 | 99.53 |
| Qatar | 3 | 0.03 | 99.56 |
| Sri Lanka | 3 | 0.03 | 99.58 |
| Uzbekistan | 3 | 0.03 | 99.61 |
| Albania | 2 | 0.02 | 99.62 |
| Bahrain | 2 | 0.02 | 99.64 |
| Brunei | 2 | 0.02 | 99.66 |
| El Salvador | 2 | 0.02 | 99.67 |
| Eritrea | 2 | 0.02 | 99.69 |
| Honduras | 2 | 0.02 | 99.71 |
| Iraq | 2 | 0.02 | 99.72 |
| Maldives | 2 | 0.02 | 99.74 |
| Netherlands Antilles | 2 | 0.02 | 99.76 |
| New Caledonia | 2 | 0.02 | 99.77 |
| Angola | 1 | 0.01 | 99.78 |
| Bahamas | 1 | 0.01 | 99.79 |
| Bangladesh | 1 | 0.01 | 99.80 |
| Bolivia | 1 | 0.01 | 99.81 |
| Bosnia and Herzegovina | 1 | 0.01 | 99.82 |
| British Virgin Islands | 1 | 0.01 | 99.82 |
| Cambodia | 1 | 0.01 | 99.83 |
| Cameroon | 1 | 0.01 | 99.84 |
| Cayman Islands | 1 | 0.01 | 99.85 |
| Djibouti | 1 | 0.01 | 99.86 |
| French Polynesia | 1 | 0.01 | 99.87 |
| Georgia | 1 | 0.01 | 99.87 |
| Haiti | 1 | 0.01 | 99.88 |
| Libya | 1 | 0.01 | 99.89 |
| Liechtenstein | 1 | 0.01 | 99.90 |
| Macedonia | 1 | 0.01 | 99.91 |
| Monaco | 1 | 0.01 | 99.92 |
| Myanmar | 1 | 0.01 | 99.92 |
| Niger | 1 | 0.01 | 99.93 |
| North Korea | 1 | 0.01 | 99.94 |
| Paraguay | 1 | 0.01 | 99.95 |
| Puerto Rico | 1 | 0.01 | 99.96 |
| Saint Kitts and Nevis | 1 | 0.01 | 99.97 |
| Somalia | 1 | 0.01 | 99.97 |
| Trinidad and Tobago | 1 | 0.01 | 99.98 |
| Yemen | 1 | 0.01 | 99.99 |
| Zambia | 1 | 0.01 | 100.00 |

SM Table 2. Performance metrics of NamSor in the marathon cohort (n = 11,999). Data are shown for the overall sample, across probability thresholds, and after exclusion of participants from Asia and China, with and without country information for gender assignment.

| Condition | n | errorCoded | errorcodedWithoutNA | naCoded | errorGenderBias |
| --- | --- | --- | --- | --- | --- |
| Without using country information |  |  |  |  |  |
| Whole sample | 11,999 | 0.0481 | 0.0481 | 0 | -0.0028 |
| ≥60% threshold | 11,679 | 0.0618 | 0.0361 | 0.0267 | -0.0041 |
| ≥70% threshold | 11,180 | 0.0928 | 0.0264 | 0.0683 | -0.0033 |
| ≥80% threshold | 10,596 | 0.1339 | 0.0193 | 0.1169 | -0.0021 |
| ≥90% threshold | 9,497 | 0.2171 | 0.0108 | 0.2085 | -0.0012 |
| Excluding participants from Asia | 7,902 | 0.0067 | 0.0067 | 0 | -0.0029 |
| Excluding participants from China | 9,947 | 0.0100 | 0.0100 | 0 | -0.0045 |
| Using country information |  |  |  |  |  |
| Whole sample | 11,999 | 0.0478 | 0.0478 | 0 | -0.0024 |
| ≥60% threshold | 11,707 | 0.0597 | 0.0362 | 0.0243 | -0.0022 |
| ≥70% threshold | 11,325 | 0.0822 | 0.0275 | 0.0562 | -0.0030 |
| ≥80% threshold | 11,042 | 0.1021 | 0.0243 | 0.0798 | -0.0020 |
| ≥90% threshold | 10,618 | 0.1322 | 0.0193 | 0.1151 | 0.0012 |
| Excluding participants from Asia | 7,902 | 0.0065 | 0.0065 | 0 | -0.0032 |
| Excluding participants from China | 9,947 | 0.0095 | 0.0095 | 0 | -0.0046 |

Percentage threshold indicates the minimum probability required by NamSor for assigning a gender classification; predictions below this confidence threshold were coded as unclassified.

SM Table 3. Performance metrics of Gender API in the marathon cohort (n = 11,999). Data are shown for the overall sample, across probability thresholds, and after exclusion of participants from Asia and China, with and without country information for gender assignment.

| Condition | n | errorCoded | errorcodedWithoutNA | naCoded | errorGenderBias |
| --- | --- | --- | --- | --- | --- |
| Without using country information |  |  |  |  |  |
| Whole sample | 11,962 | 0.0867 | 0.0838 | 0.0031 | -0.0370 |
| ≥60% threshold | 11,473 | 0.1088 | 0.0679 | 0.0438 | -0.0339 |
| ≥70% threshold | 10,763 | 0.1453 | 0.0472 | 0.1030 | -0.0242 |
| ≥80% threshold | 10,059 | 0.1873 | 0.0306 | 0.1617 | -0.0149 |
| ≥90% threshold | 9,011 | 0.2623 | 0.0176 | 0.2490 | -0.0108 |
| Excluding participants from Asia | 7,888 | 0.0352 | 0.0335 | 0.0018 | -0.0119 |
| Excluding participants from China | 9,915 | 0.0473 | 0.0442 | 0.0032 | -0.0173 |
| Using country information |  |  |  |  |  |
| Whole sample | 11,955 | 0.0753 | 0.0719 | 0.0037 | -0.0328 |
| ≥60% threshold | 11,593 | 0.0927 | 0.0609 | 0.0338 | -0.0300 |
| ≥70% threshold | 11,006 | 0.1229 | 0.0438 | 0.0828 | -0.0196 |
| ≥80% threshold | 10,262 | 0.1685 | 0.0278 | 0.1448 | -0.0120 |
| ≥90% threshold | 9,205 | 0.2474 | 0.0190 | 0.2329 | -0.0088 |
| Excluding participants from Asia | 7,880 | 0.0295 | 0.0268 | 0.0028 | -0.0070 |
| Excluding participants from China | 9,907 | 0.0361 | 0.0322 | 0.0040 | -0.0116 |

Percentage threshold indicates the minimum probability required by Gender API for assigning a gender classification; predictions below this confidence threshold were coded as unclassified.

SM Table 4. Performance metrics of genderize.io in the marathon cohort (n = 11,999). Data are shown for the overall sample, across probability thresholds, and after exclusion of participants from Asia and China, with and without country information for gender assignment.

| Condition | n | errorCoded | errorcodedWithoutNA | naCoded | errorGenderBias |
| --- | --- | --- | --- | --- | --- |
| Without using country information |  |  |  |  |  |
| Whole sample | 11,984 | 0.0798 | 0.0786 | 0.0013 | -0.0152 |
| ≥60% threshold | 11,455 | 0.1018 | 0.0591 | 0.0453 | -0.0106 |
| ≥70% threshold | 10,875 | 0.1329 | 0.0433 | 0.0937 | -0.0106 |
| ≥80% threshold | 10,344 | 0.1653 | 0.0317 | 0.1379 | -0.0093 |
| ≥90% threshold | 9,581 | 0.2167 | 0.0190 | 0.2015 | -0.0069 |
| Excluding participants from Asia | 7,897 | 0.0315 | 0.0309 | 0.0006 | -0.0063 |
| Excluding participants from China | 9,934 | 0.0426 | 0.0414 | 0.0013 | -0.0088 |
| Using country information |  |  |  |  |  |
| Whole sample | 11,925 | 0.0753 | 0.0695 | 0.0062 | -0.0177 |
| ≥60% threshold | 11,447 | 0.0968 | 0.0533 | 0.0460 | -0.0176 |
| ≥70% threshold | 11,034 | 0.1199 | 0.0423 | 0.0804 | -0.0148 |
| ≥80% threshold | 10,643 | 0.1438 | 0.0347 | 0.1130 | -0.0129 |
| ≥90% threshold | 10,101 | 0.1791 | 0.0248 | 0.1582 | -0.0100 |
| Excluding participants from Asia | 7,860 | 0.0290 | 0.0238 | 0.0053 | -0.0078 |
| Excluding participants from China | 9,879 | 0.0369 | 0.0303 | 0.0068 | -0.0078 |

Percentage threshold indicates the minimum probability required by genderize.io for assigning a gender classification; predictions below this confidence threshold were coded as unclassified.

SM Table 5. Performance metrics of NamSor, Gender API, and genderize.io by region of nationality in the marathon cohort (n = 11,999), with and without country information for gender assignment.

| Condition | n | errorCoded | errorcodedWithoutNA | naCoded | errorGenderBias |
| --- | --- | --- | --- | --- | --- |
| Without using country information |  |  |  |  |  |
| Namsor |  |  |  |  |  |
| Europe | 4,702 | 0.0036 | 0.0036 | 0 | -0.0002 |
| Asia | 4,097 | 0.1279 | 0.1279 | 0 | -0.0024 |
| Americas | 2,601 | 0.0111 | 0.0111 | 0 | -0.0065 |
| Africa | 410 | 0.0146 | 0.0146 | 0 | -0.0146 |
| Oceania | 189 | 0.0053 | 0.0053 | 0 | 0.0053 |
| Gender API |  |  |  |  |  |
| Europe | 4,695 | 0.0238 | 0.0224 | 0.0015 | -0.0032 |
| Asia | 4,074 | 0.1860 | 0.1814 | 0.0046 | -0.0857 |
| Americas | 2,597 | 0.0419 | 0.0404 | 0.0015 | -0.0219 |
| Africa | 407 | 0.1195 | 0.1130 | 0.0073 | -0.0639 |
| Oceania | 189 | 0.0423 | 0.0423 | 0 | 0.0212 |
| Genderize.io |  |  |  |  |  |
| Europe | 4,699 | 0.0240 | 0.0234 | 0.0006 | 0.0013 |
| Asia | 4,087 | 0.1728 | 0.1708 | 0.0024 | -0.0323 |
| Americas | 2,599 | 0.0392 | 0.0385 | 0.0008 | -0.0223 |
| Africa | 410 | 0.0707 | 0.0707 | 0 | -0.0024 |
| Oceania | 189 | 0.0265 | 0.0265 | 0 | 0.0159 |
| Using country information |  |  |  |  |  |
| NamSor |  |  |  |  |  |
| Europe | 4,702 | 0.0032 | 0.0032 | 0 | -0.0002 |
| Asia | 4,097 | 0.1274 | 0.1274 | 0 | -0.0010 |
| Americas | 2,601 | 0.0104 | 0.0104 | 0 | -0.0065 |
| Africa | 410 | 0.0195 | 0.0195 | 0 | -0.0195 |
| Oceania | 189 | 0.0053 | 0.0053 | 0 | 0.0053 |
| Gender API |  |  |  |  |  |
| Europe | 4,695 | 0.0172 | 0.0158 | 0.0015 | -0.0043 |
| Asia | 4,075 | 0.1638 | 0.1593 | 0.0054 | -0.0827 |
| Americas | 2,590 | 0.0427 | 0.0386 | 0.0042 | -0.0023 |
| Africa | 406 | 0.0927 | 0.0837 | 0.0098 | -0.0739 |
| Oceania | 189 | 0.0159 | 0.0159 | 0 | 0.0053 |
| Genderize.io |  |  |  |  |  |
| Europe | 4,681 | 0.0196 | 0.0152 | 0.0045 | -0.0006 |
| Asia | 4,065 | 0.1645 | 0.1579 | 0.0078 | -0.0369 |
| Americas | 2,591 | 0.0311 | 0.0274 | 0.0038 | -0.0143 |
| Africa | 401 | 0.1098 | 0.0898 | 0.0220 | -0.0499 |
| Oceania | 187 | 0.0582 | 0.0481 | 0.0106 | -0.0053 |

SM Table 6. Performance metrics of NamSor by country of nationality (n = 11,999 participants). Data are shown for countries with at least 20 participants.

| Country | n | errorCoded | errorCodedWithoutNA | naCoded | errorGenderBias |
| --- | --- | --- | --- | --- | --- |
| China | 2052 | 0.2329 | 0.2329 | 0.0000 | 0.0058 |
| United States | 1863 | 0.0118 | 0.0118 | 0.0000 | -0.0086 |
| France | 1616 | 0.0006 | 0.0006 | 0.0000 | -0.0006 |
| Japan | 1149 | 0.0026 | 0.0026 | 0.0000 | 0.0009 |
| United Kingdom | 955 | 0.0031 | 0.0031 | 0.0000 | -0.0010 |
| Germany | 446 | 0.0045 | 0.0045 | 0.0000 | 0.0045 |
| Philippines | 231 | 0.0866 | 0.0866 | 0.0000 | -0.0866 |
| Canada | 217 | 0.0184 | 0.0184 | 0.0000 | -0.0092 |
| Russia | 189 | 0.0053 | 0.0053 | 0.0000 | 0.0053 |
| Spain | 166 | 0.0060 | 0.0060 | 0.0000 | -0.0060 |
| Australia | 155 | 0.0065 | 0.0065 | 0.0000 | 0.0065 |
| Brazil | 155 | 0.0129 | 0.0129 | 0.0000 | 0.0129 |
| Italy | 155 | 0.0000 | 0.0000 | 0.0000 | 0.0000 |
| Mexico | 150 | 0.0000 | 0.0000 | 0.0000 | 0.0000 |
| Ireland | 148 | 0.0068 | 0.0068 | 0.0000 | 0.0068 |
| South Africa | 133 | 0.0226 | 0.0226 | 0.0000 | -0.0226 |
| Netherlands | 128 | 0.0156 | 0.0156 | 0.0000 | 0.0000 |
| Norway | 122 | 0.0164 | 0.0164 | 0.0000 | -0.0164 |
| Poland | 117 | 0.0171 | 0.0171 | 0.0000 | 0.0171 |
| India | 100 | 0.0000 | 0.0000 | 0.0000 | 0.0000 |
| Ethiopia | 96 | 0.0000 | 0.0000 | 0.0000 | 0.0000 |
| Belgium | 94 | 0.0000 | 0.0000 | 0.0000 | 0.0000 |
| Switzerland | 81 | 0.0123 | 0.0123 | 0.0000 | -0.0123 |
| Kenya | 76 | 0.0132 | 0.0132 | 0.0000 | -0.0132 |
| Iran | 71 | 0.0000 | 0.0000 | 0.0000 | 0.0000 |
| United Arab Emirates | 71 | 0.0141 | 0.0141 | 0.0000 | -0.0141 |
| Sweden | 64 | 0.0000 | 0.0000 | 0.0000 | 0.0000 |
| Colombia | 61 | 0.0000 | 0.0000 | 0.0000 | 0.0000 |
| Denmark | 56 | 0.0000 | 0.0000 | 0.0000 | 0.0000 |
| Hong Kong | 52 | 0.0385 | 0.0385 | 0.0000 | -0.0385 |
| Austria | 46 | 0.0000 | 0.0000 | 0.0000 | 0.0000 |
| Kazakhstan | 41 | 0.0000 | 0.0000 | 0.0000 | 0.0000 |
| Portugal | 40 | 0.0000 | 0.0000 | 0.0000 | 0.0000 |
| South Korea | 37 | 0.1081 | 0.1081 | 0.0000 | 0.1081 |
| Taiwan | 37 | 0.0270 | 0.0270 | 0.0000 | -0.0270 |
| Argentina | 33 | 0.0303 | 0.0303 | 0.0000 | -0.0303 |
| New Zealand | 31 | 0.0000 | 0.0000 | 0.0000 | 0.0000 |
| Chile | 29 | 0.0000 | 0.0000 | 0.0000 | 0.0000 |
| Lithuania | 29 | 0.0000 | 0.0000 | 0.0000 | 0.0000 |
| Finland | 28 | 0.0000 | 0.0000 | 0.0000 | 0.0000 |
| Greece | 27 | 0.0000 | 0.0000 | 0.0000 | 0.0000 |
| Israel | 25 | 0.0000 | 0.0000 | 0.0000 | 0.0000 |
| Romania | 25 | 0.0000 | 0.0000 | 0.0000 | 0.0000 |
| Czech Republic | 24 | 0.0000 | 0.0000 | 0.0000 | 0.0000 |
| Egypt | 24 | 0.0000 | 0.0000 | 0.0000 | 0.0000 |
| Lebanon | 24 | 0.0000 | 0.0000 | 0.0000 | 0.0000 |
| Morocco | 24 | 0.0417 | 0.0417 | 0.0000 | -0.0417 |
| Costa Rica | 23 | 0.0000 | 0.0000 | 0.0000 | 0.0000 |
| Slovak Republic | 22 | 0.0000 | 0.0000 | 0.0000 | 0.0000 |
| Malaysia | 21 | 0.0952 | 0.0952 | 0.0000 | 0.0000 |
| Hungary | 20 | 0.0500 | 0.0500 | 0.0000 | -0.0500 |
| Singapore | 20 | 0.3000 | 0.3000 | 0.0000 | -0.2000 |
| Ukraine | 20 | 0.0000 | 0.0000 | 0.0000 | 0.0000 |

SM Table 7. Performance metrics of Gender API by country of nationality (n = 11,999 participants). Data are shown for countries with at least 20 participants.

| Country | n | errorCoded | errorCodedWithoutNA | naCoded | errorGenderBias |
| --- | --- | --- | --- | --- | --- |
| China | 2052 | 0.2778 | 0.2760 | 0.0024 | -0.1324 |
| United States | 1863 | 0.0462 | 0.0446 | 0.0016 | -0.0242 |
| France | 1616 | 0.0167 | 0.0161 | 0.0006 | 0.0000 |
| Japan | 1149 | 0.0748 | 0.0748 | 0.0000 | -0.0139 |
| United Kingdom | 955 | 0.0325 | 0.0314 | 0.0010 | -0.0126 |
| Germany | 446 | 0.0202 | 0.0180 | 0.0022 | 0.0000 |
| Philippines | 231 | 0.1515 | 0.1211 | 0.0346 | -0.1031 |
| Canada | 217 | 0.0691 | 0.0691 | 0.0000 | -0.0323 |
| Russia | 189 | 0.0159 | 0.0106 | 0.0053 | 0.0000 |
| Spain | 166 | 0.0241 | 0.0241 | 0.0000 | -0.0120 |
| Australia | 155 | 0.0452 | 0.0452 | 0.0000 | 0.0194 |
| Brazil | 155 | 0.0258 | 0.0258 | 0.0000 | -0.0129 |
| Italy | 155 | 0.0323 | 0.0323 | 0.0000 | 0.0065 |
| Mexico | 150 | 0.0133 | 0.0067 | 0.0067 | -0.0067 |
| Ireland | 148 | 0.0405 | 0.0405 | 0.0000 | 0.0135 |
| South Africa | 133 | 0.0902 | 0.0902 | 0.0000 | -0.0602 |
| Netherlands | 128 | 0.0469 | 0.0469 | 0.0000 | -0.0156 |
| Norway | 122 | 0.0410 | 0.0410 | 0.0000 | -0.0082 |
| Poland | 117 | 0.0085 | 0.0085 | 0.0000 | -0.0085 |
| India | 100 | 0.0200 | 0.0200 | 0.0000 | 0.0000 |
| Ethiopia | 96 | 0.2813 | 0.2660 | 0.0208 | -0.2021 |
| Belgium | 94 | 0.0000 | 0.0000 | 0.0000 | 0.0000 |
| Switzerland | 81 | 0.0494 | 0.0494 | 0.0000 | 0.0000 |
| Kenya | 76 | 0.0395 | 0.0395 | 0.0000 | 0.0395 |
| Iran | 71 | 0.0000 | 0.0000 | 0.0000 | 0.0000 |
| United Arab Emirates | 71 | 0.0000 | 0.0000 | 0.0000 | 0.0000 |
| Sweden | 64 | 0.0156 | 0.0156 | 0.0000 | 0.0156 |
| Colombia | 61 | 0.0000 | 0.0000 | 0.0000 | 0.0000 |
| Denmark | 56 | 0.0000 | 0.0000 | 0.0000 | 0.0000 |
| Hong Kong | 52 | 0.4038 | 0.4038 | 0.0000 | -0.3269 |
| Austria | 46 | 0.0000 | 0.0000 | 0.0000 | 0.0000 |
| Kazakhstan | 41 | 0.1220 | 0.0270 | 0.0976 | -0.0270 |
| Portugal | 40 | 0.0000 | 0.0000 | 0.0000 | 0.0000 |
| South Korea | 37 | 0.1351 | 0.0857 | 0.0541 | -0.0857 |
| Taiwan | 37 | 0.2973 | 0.2973 | 0.0000 | -0.1892 |
| Argentina | 33 | 0.0303 | 0.0303 | 0.0000 | -0.0303 |
| New Zealand | 31 | 0.0323 | 0.0323 | 0.0000 | 0.0323 |
| Chile | 29 | 0.0000 | 0.0000 | 0.0000 | 0.0000 |
| Lithuania | 29 | 0.0345 | 0.0000 | 0.0345 | 0.0000 |
| Finland | 28 | 0.0714 | 0.0714 | 0.0000 | 0.0714 |
| Greece | 27 | 0.0741 | 0.0385 | 0.0370 | -0.0385 |
| Israel | 25 | 0.0800 | 0.0800 | 0.0000 | 0.0000 |
| Romania | 25 | 0.0000 | 0.0000 | 0.0000 | 0.0000 |
| Czech Republic | 24 | 0.0000 | 0.0000 | 0.0000 | 0.0000 |
| Egypt | 24 | 0.0000 | 0.0000 | 0.0000 | 0.0000 |
| Lebanon | 24 | 0.0000 | 0.0000 | 0.0000 | 0.0000 |
| Morocco | 24 | 0.0417 | 0.0417 | 0.0000 | -0.0417 |
| Costa Rica | 23 | 0.0000 | 0.0000 | 0.0000 | 0.0000 |
| Slovak Republic | 22 | 0.0000 | 0.0000 | 0.0000 | 0.0000 |
| Malaysia | 21 | 0.1905 | 0.1905 | 0.0000 | -0.0952 |
| Hungary | 20 | 0.0500 | 0.0500 | 0.0000 | -0.0500 |
| Singapore | 20 | 0.4000 | 0.4000 | 0.0000 | -0.3000 |
| Ukraine | 20 | 0.0500 | 0.0500 | 0.0000 | 0.0500 |

SM Table 8. Performance metrics of genderize.io by country of nationality (n = 11,999 participants). Data are shown for countries with at least 20 participants.

| Country | n | errorCoded | errorCodedWithoutNA | naCoded | errorGenderBias |
| --- | --- | --- | --- | --- | --- |
| China | 2052 | 0.2597 | 0.2590 | 0.0010 | -0.0463 |
| United States | 1863 | 0.0424 | 0.0419 | 0.0005 | -0.0290 |
| France | 1616 | 0.0217 | 0.0211 | 0.0006 | 0.0062 |
| Japan | 1149 | 0.0688 | 0.0688 | 0.0000 | 0.0044 |
| United Kingdom | 955 | 0.0251 | 0.0251 | 0.0000 | -0.0188 |
| Germany | 446 | 0.0179 | 0.0179 | 0.0000 | 0.0090 |
| Philippines | 231 | 0.1169 | 0.1169 | 0.0000 | -0.0823 |
| Canada | 217 | 0.0599 | 0.0556 | 0.0046 | -0.0185 |
| Russia | 189 | 0.0053 | 0.0053 | 0.0000 | 0.0053 |
| Spain | 166 | 0.0422 | 0.0422 | 0.0000 | -0.0060 |
| Australia | 155 | 0.0323 | 0.0323 | 0.0000 | 0.0194 |
| Brazil | 155 | 0.0452 | 0.0452 | 0.0000 | -0.0065 |
| Italy | 155 | 0.0387 | 0.0387 | 0.0000 | 0.0258 |
| Mexico | 150 | 0.0000 | 0.0000 | 0.0000 | 0.0000 |
| Ireland | 148 | 0.0203 | 0.0203 | 0.0000 | -0.0068 |
| South Africa | 133 | 0.0752 | 0.0752 | 0.0000 | -0.0451 |
| Netherlands | 128 | 0.0313 | 0.0236 | 0.0078 | -0.0079 |
| Norway | 122 | 0.0492 | 0.0492 | 0.0000 | -0.0164 |
| Poland | 117 | 0.0256 | 0.0256 | 0.0000 | 0.0256 |
| India | 100 | 0.0400 | 0.0400 | 0.0000 | 0.0000 |
| Ethiopia | 96 | 0.1146 | 0.1146 | 0.0000 | 0.0313 |
| Belgium | 94 | 0.0106 | 0.0106 | 0.0000 | 0.0106 |
| Switzerland | 81 | 0.0370 | 0.0370 | 0.0000 | 0.0123 |
| Kenya | 76 | 0.0395 | 0.0395 | 0.0000 | 0.0395 |
| Iran | 71 | 0.0141 | 0.0141 | 0.0000 | 0.0141 |
| United Arab Emirates | 71 | 0.0000 | 0.0000 | 0.0000 | 0.0000 |
| Sweden | 64 | 0.0469 | 0.0469 | 0.0000 | 0.0469 |
| Colombia | 61 | 0.0000 | 0.0000 | 0.0000 | 0.0000 |
| Denmark | 56 | 0.0000 | 0.0000 | 0.0000 | 0.0000 |
| Hong Kong | 52 | 0.3654 | 0.3654 | 0.0000 | -0.2885 |
| Austria | 46 | 0.0000 | 0.0000 | 0.0000 | 0.0000 |
| Kazakhstan | 41 | 0.0732 | 0.0256 | 0.0488 | -0.0256 |
| Portugal | 40 | 0.0000 | 0.0000 | 0.0000 | 0.0000 |
| South Korea | 37 | 0.1622 | 0.1389 | 0.0270 | -0.0278 |
| Taiwan | 37 | 0.1892 | 0.1892 | 0.0000 | -0.0270 |
| Argentina | 33 | 0.0303 | 0.0303 | 0.0000 | -0.0303 |
| New Zealand | 31 | 0.0000 | 0.0000 | 0.0000 | 0.0000 |
| Chile | 29 | 0.0000 | 0.0000 | 0.0000 | 0.0000 |
| Lithuania | 29 | 0.0000 | 0.0000 | 0.0000 | 0.0000 |
| Finland | 28 | 0.1429 | 0.1429 | 0.0000 | 0.1429 |
| Greece | 27 | 0.0741 | 0.0385 | 0.0370 | -0.0385 |
| Israel | 25 | 0.0800 | 0.0800 | 0.0000 | 0.0000 |
| Romania | 25 | 0.0000 | 0.0000 | 0.0000 | 0.0000 |
| Czech Republic | 24 | 0.0000 | 0.0000 | 0.0000 | 0.0000 |
| Egypt | 24 | 0.0000 | 0.0000 | 0.0000 | 0.0000 |
| Lebanon | 24 | 0.0000 | 0.0000 | 0.0000 | 0.0000 |
| Morocco | 24 | 0.0417 | 0.0417 | 0.0000 | -0.0417 |
| Costa Rica | 23 | 0.0000 | 0.0000 | 0.0000 | 0.0000 |
| Slovak Republic | 22 | 0.0000 | 0.0000 | 0.0000 | 0.0000 |
| Malaysia | 21 | 0.2381 | 0.2381 | 0.0000 | -0.1429 |
| Hungary | 20 | 0.0500 | 0.0500 | 0.0000 | -0.0500 |
| Singapore | 20 | 0.5000 | 0.5000 | 0.0000 | -0.2000 |
| Ukraine | 20 | 0.0500 | 0.0500 | 0.0000 | 0.0500 |
